# Substitution at the C-3 Position of Catechins Has an Influence on the Binding Affinities against Serum Albumin

**DOI:** 10.3390/molecules22020314

**Published:** 2017-02-18

**Authors:** Masaki Ikeda, Manabu Ueda-Wakagi, Kaori Hayashibara, Rei Kitano, Masaya Kawase, Kunihiro Kaihatsu, Nobuo Kato, Yoshitomo Suhara, Naomi Osakabe, Hitoshi Ashida

**Affiliations:** 1 Graduate School of Agricultural Science, Kobe University, 1-1 Rokkodai, Nada-ku, Kobe, Hyogo 657-8501, Japan; ike.glr.sindai@gmail.com (M.I.); sssuuntto@gmail.com (K.H.); kitano_rei@fancl.co.jp (R.K.); 2 National Agriculture and Food Research Organization, National Food Research Institute, Tsukuba, Ibaraki 305-8642, Japan; mana5998bu@affrc.go.jp; 3 Department of Bioscience, Nagahama Institute of Bio-Science and Technology, 1266 Tamura-cho, Nagahama, Shiga 526-0829, Japan; kawase203@yahoo.co.jp; 4 Department of Organic Fine Chemicals, The Institute of Scientific and Industrial Research, Osaka University, 8-1, Mihogaoka, Ibaraki, Osaka 567-0047, Japan; kunihiro@sanken.osaka-u.ac.jp (K.K.); kato-n@sanken.osaka-u.ac.jp (N.K.); 5 Department of Bioscience and Engineering, College of Systems Engineering and Science, Shibaura Institute of Technology, 307 Fukasaku, Minuma-ku, Saitama 337-8570, Japan; suhara@sic.shibaura-it.ac.jp (Y.S.); nao-osa@sic.shibaura-it.ac.jp (N.O.)

**Keywords:** catechin, serum albumin, interaction, docking study, fluorescence analysis

## Abstract

It is known that catechins interact with the tryptophan (Trp) residue at the drug-binding site of serum albumin. In this study, we used catechin derivatives to investigate which position of the catechin structure strongly influences the binding affinity against bovine serum albumin (BSA) and human serum albumin (HSA). A docking simulation showed that (−)-epigallocatechin gallate (EGCg) interacted with both Trp residues of BSA (one at drug-binding site I and the other on the molecular surface), mainly by π–π stacking. Fluorescence analysis showed that EGCg and substituted EGCg caused a red shift of the peak wavelength of Trp similarly to warfarin (a drug-binding site I-specific compound), while 3-*O*-acyl-catechins caused a blue shift. To evaluate the binding affinities, the quenching constants were determined by the Stern–Volmer equation. A gallate ester at the C-3 position increased the quenching constants of the catechins. Against BSA, acyl substitution increased the quenching constant proportionally to the carbon chain lengths of the acyl group, whereas methyl substitution decreased the quenching constant. Against HSA, neither acyl nor methyl substitution affected the quenching constant. In conclusion, substitution at the C-3 position of catechins has an important influence on the binding affinity against serum albumin.

## 1. Introduction

Catechins are natural active compounds contained in foods such as tea, grapes, chocolate, apples, and berries [1]. Catechins possess two benzene rings (the A- and B-rings) and a dihydropyran heterocycle (the C-ring) with a hydroxyl group at the C-3 position. Catechins are classified into *cis*- and *trans*-types from the configuration of the two hydrogens at the C-2 and C-3 positions on the C-ring. Gallate-type catechins have a gallate ester at the C-3 position. (−)-Epigallocatechin gallate (EGCg) is the most abundant catechin in tea leaves. Methylated catechins such as epigallocatechin-3-*O*-methyl gallate are abundant in Benifuuki (*Camellia sinensis* L.), a tea cultivar [2]. 

Catechins possess various beneficial health effects, including antioxidant [3], anti-inflammatory [4], anti-cancer [5], and anti-diabetic activities [6]. Our previous study showed that tea catechins—except for (+)-catechin [(+)-C] and (−)-catechin gallate—significantly increased glucose uptake activity in skeletal muscle cells [7]. We also reported that the addition of an acyl group to the C-3 position of (−)-epicatechin ((−)-EC) increased its affinity for the lipid bilayer membrane and promoted glucose transporter type 4 translocation through activation of the phosphoinositide 3-kinase signaling pathway in L6 myotubes [8]. Moreover, we demonstrated that gallate-type catechins reduced insulin-induced glucose uptake activity in 3T3-L1 adipocytes, whereas non-gallate-type catechins did not [9]. These results suggest that the functions of catechins are sensitive to their chemical structure, including their spatial configuration and modification by functional groups. Moreover, it is important to clarify the interactions between catechins and their target proteins to elucidate the mechanism of their beneficial health effects.

Serum albumin is the major soluble protein constituent of the circulatory system. After intake of dietary flavonoids, the incorporated flavonoids and their metabolites first interact with albumin protein in blood to enable transport of the compounds to various tissues of the body. Bovine serum albumin (BSA) is one of the most extensively studied proteins because of its structural homology with human serum albumin (HSA) [10]. BSA interacts with a wide range of chemicals, such as sanguinarine, quercetin, rutin, gemcitabine hydrochloride, and proflavin [11,12,13,14]. For the interaction of BSA with dietary flavonoids, the binding efficiency is governed by the structural arrangement, and the resonance between the A- and B-rings is reported to be essential for the biological activity of these flavonoids. 

To measure the binding affinity of chemicals or biological agents to proteins, fluorescence analysis based on the fluorescent properties of tryptophan (Trp) residue(s) in the target protein is a useful tool, because Trp fluorescence is remarkably sensitive to the polarity of the environment [15,16,17]. Möller and Denicola measured fluorescence quenching of Trp by iodide using free Trp, ovalbumin, and BSA [16]. Krafft et al. determined the binding affinities of peptides derived from the proteins Raf-1 and KSR-1 to small unilamellar vesicles containing phosphatidic acid [17]. Sonveaux et al. analyzed ligand-mediated tertiary structural changes of reconstituted P-glycoprotein by a Trp fluorescence quenching analysis [18]. Matveeva et al. measured the binding of the naphtholurea inhibitor XIIa to the enzyme epoxide hydrolase [19]. The success of these studies strongly suggests that fluorescence analysis is applicable to estimate the binding affinity of catechins against serum albumin. 

Therefore, in this study, we used docking simulations and Trp fluorescence quenching analysis to investigate which position of the catechin structure regulates the binding affinity against BSA and HSA. The structure–binding relationship was explored by using various catechin derivatives; i.e., acyl- and methyl-epigallocatechin gallate (methyl-EGCg) and acyl-catechins (formed by the addition of acyl groups with carbon chain lengths from 6 to 18 at the C-3 position). 

## 2. Results

### 2.1. Interaction between BSA and EGCg and Its Derivatives 

**Figure 1:**
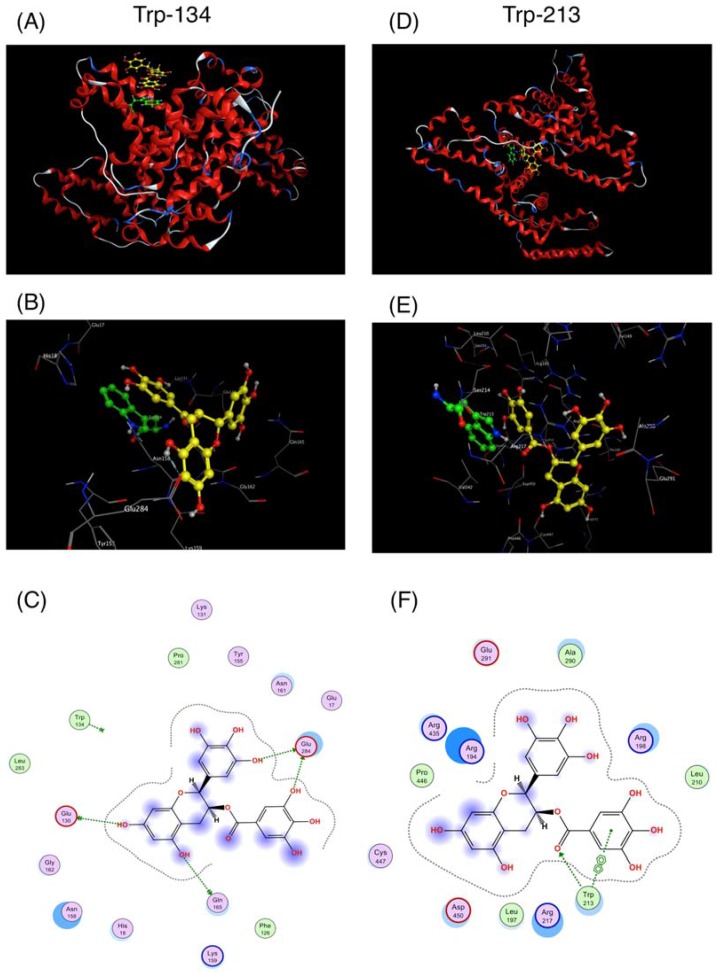
Interaction of epigallocatechin gallate (EGCg) with Trp residues in bovine serum albumin (BSA) by docking simulation. The simulation was carried out using the Docking program of the Molecular Operating Environment (MOE) suite. 3D model of the docking pose of EGCg (yellow) against Trp-134 at the protein surface (**A**) and against Trp-213 in the drug-binding site I (**D**) of BSA (red cartoon). Enlarged views of (A,D) are shown in (**B**,**E**), respectively. 2D models of (B,E) are shown in (**C**,**F**), respectively.

BSA has two Trp residues (Trp-134 and Trp-213); Trp-134 is located on the surface of the molecule, and Trp-213 is located within drug-binding site I [20]. To examine the interaction between BSA and EGCg, a 3D docking simulation was carried out (Figure 1). From the results of 3D-modeling simulation, EGCg was observed to interact with both Trp residues (Figure 1A,D). Further inspection showed that the interaction with both Trp-134 and Trp-213 occurred at the gallate ester moiety (D-ring) of EGCg by π–π stacking (Figure 1B,E). Using the lowest calculated binding energy, the affinity of EGCg against Trp-134 and Trp-213 was −26.88 kJ/mol and −30.32 kJ/mol, respectively. Moreover, EGCg also interacted with Glu-130, Glu-165, and Glu-284, located on the surface of BSA, through hydrogen bonds (Figure 1C). In the drug-binding site, a hydrogen bond was also observed between EGCg and Trp-213 (Figure 1F), which may explain why the affinity against Trp-213 was calculated to be stronger than that against Trp-134. 

**Figure 2:**
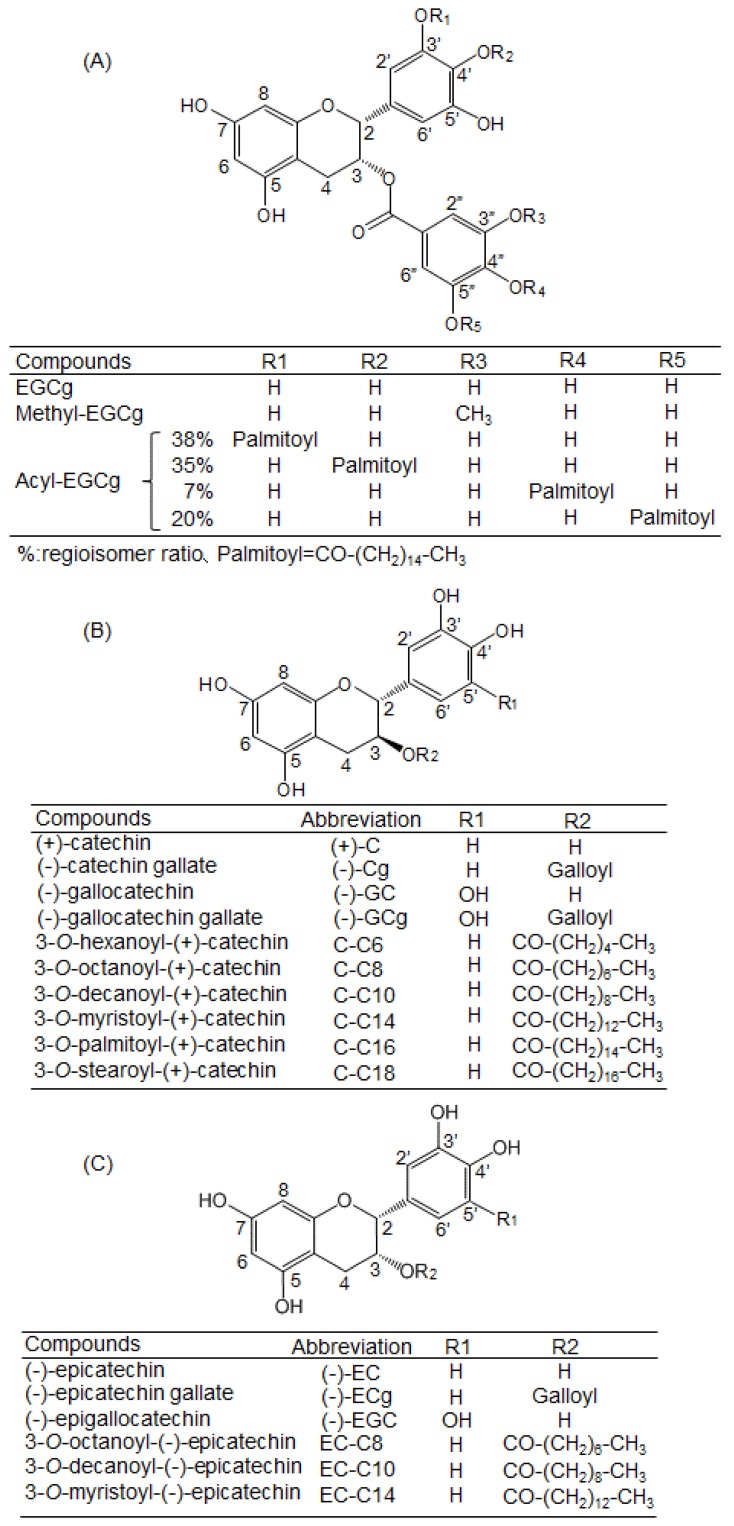
Structures of (**A**) EGCg; (**B**) (+)-C; (**C**) (−)-EC, and their derivatives.

**Figure 3:**
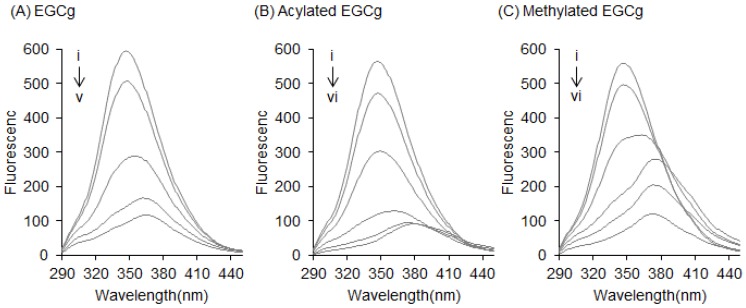
Effects of EGCg and its derivatives on the fluorescence of BSA. Fluorescence spectra of 0.3 mg/mL BSA with various concentrations of (**A**) EGCg; (**B**) acylated EGCg; and (**C**) methylated EGCg were measured at an excitation wavelength of 280 nm. Symbols i to vi show the spectra of BSA with increasing quencher concentrations of 0, 1, 5, 12.5, 25, and 50 µM, respectively.

Fluorescence measurements give information about the molecular environment of a chemical interacting with a target protein; specifically, the fluorescence of a protein can be quenched by the interaction between its Trp residues and a ligand [21,22]. Herein, BSA showed a strong fluorescence peak around 345 nm—mainly arising from its Trp residues—when the excitation wavelength was set at 280 nm. The fluorescence quenching of the Trp residues in BSA was measured after treatment with EGCg and its derivatives to estimate their binding affinity against BSA. The structures of the studied compounds are shown in Figure 2. After BSA (0.3 mg/mL) was treated with various concentrations of EGCg (1–25 µM), the fluorescence intensity of the Trp residues decreased in a dose-dependent manner (Figure 3A). The peak wavelength was shifted to the longer side (red-shifted) by EGCg. The fluorescence spectra of another natural catechin was also measured (data not shown). Figure 3B,C show the fluorescence spectra of acylated EGCg and methylated EGCg. These EGCg derivatives quenched the fluorescence of BSA in the same manner as EGCg, with a red shift of the peak wavelength. Neither EGCg nor its derivatives showed any intrinsic fluorescence under our experimental conditions (data not shown).

### 2.2. Stern–Volmer Quenching Constants of Catechins and EGCg Derivatives 

**Figure 4:**
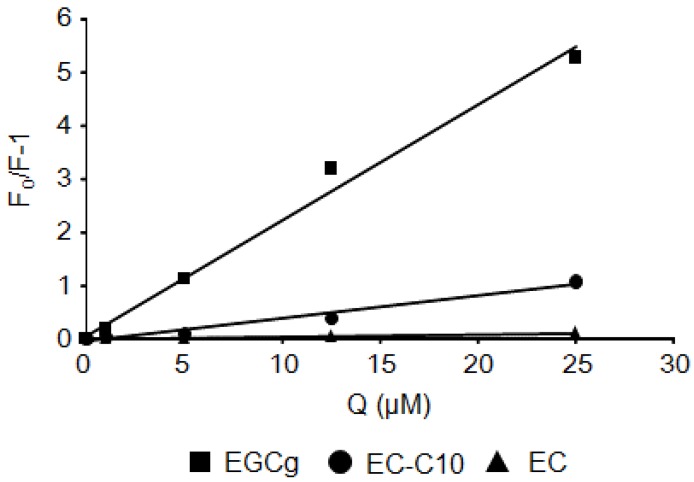
Stern–Volmer plots of catechins and BSA. Representative results for EGCg, EC-C10, and (−)-EC are shown.

From the data obtained by fluorescence analysis, the Stern–Volmer quenching constants (Kq values) were calculated by the Stern–Volmer relationship. Representative Stern–Volmer plots are shown in Figure 4. The Kq values were determined from the slopes of the plots, and are summarized in Table 1. In the case of BSA, the Kq value of EGCg was the largest among the eight natural catechins. The presence of a gallate ester moiety at the C-3 position increased the Kq values of the catechins against BSA. In contrast, differences in either the chemical substituents or the stereochemistry of the B-ring did not affect their Kq values. For the EGCg derivatives, acyl-EGCg increased the Kq value compared with the parent compound, while methyl-EGCg decreased the Kq value.

### 2.3. Interaction between BSA and 3-O-acyl-Catechins

**Figure 5:**
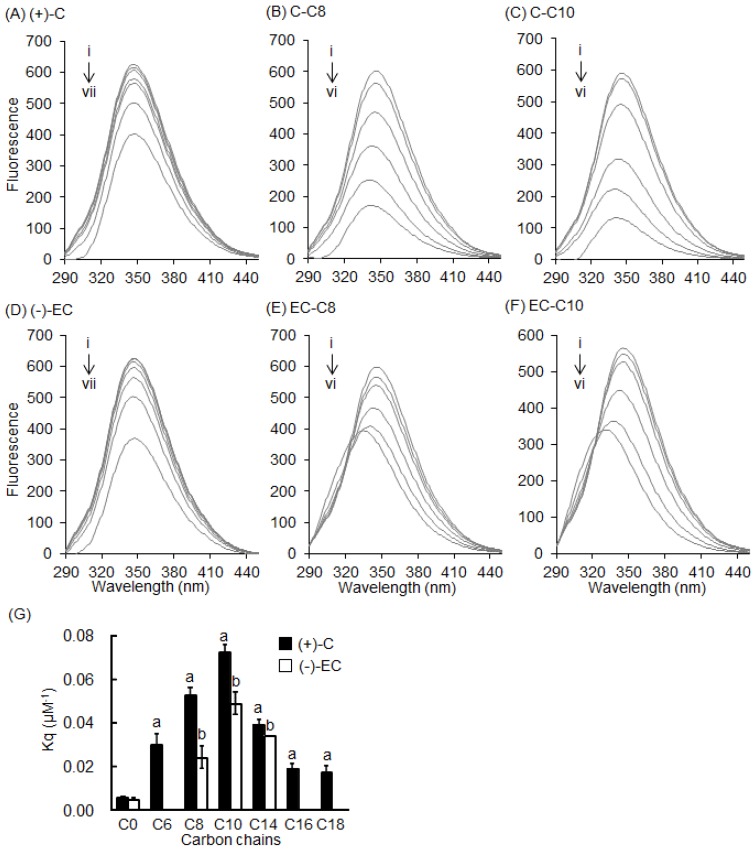
Effects of catechins and 3-*O*-acyl-catechins on fluorescence of BSA. Fluorescence spectra of 0.3 mg/mL BSA with various concentrations of (**A**) (+)-C; (**B**) C-C8; (**C**) C-C10; (**D**) (−)-EC; (**E**) EC-C8; and (**F**) EC-C10 were measured at an excitation wavelength of 280 nm. Symbols i to vii show the spectra of BSA with increasing quencher concentrations of 0, 1, 5, 12.5, 25, 50, and 100 µM, respectively; (**G**) Relationship between Kq value and chain length of 3-*O*-acyl-catechins.

To confirm the importance of the C-3 position of catechins, 3-*O*-acyl-catechins were studied. Acyl-(+)-C (C8 and C10) greatly decreased the fluorescence intensity of BSA compared with (+)-C (Figure 5A–C). Similar results were observed with acyl-(−)-EC (Figure 5D–F). The peak wavelength was shifted to the shorter side (blue-shifted) by the acyl-catechins. The fluorescence intensity of BSA treated with a series of 3-*O*-acyl-catechins was also measured, and their Kq values were calculated (Figure 5G). All the acyl-catechins significantly increased the Kq values compared with the parent compound (C0). The optimal chain length of the acyl group for quenching Trp residues in BSA was C10. This implies that the binding mode of acyl-catechins with short acyl groups (≤C10) was different from those with long acyl groups (>C10). 

### 2.4. Interaction between HSA and Catechins and Their Derivatives

**Figure 6:**
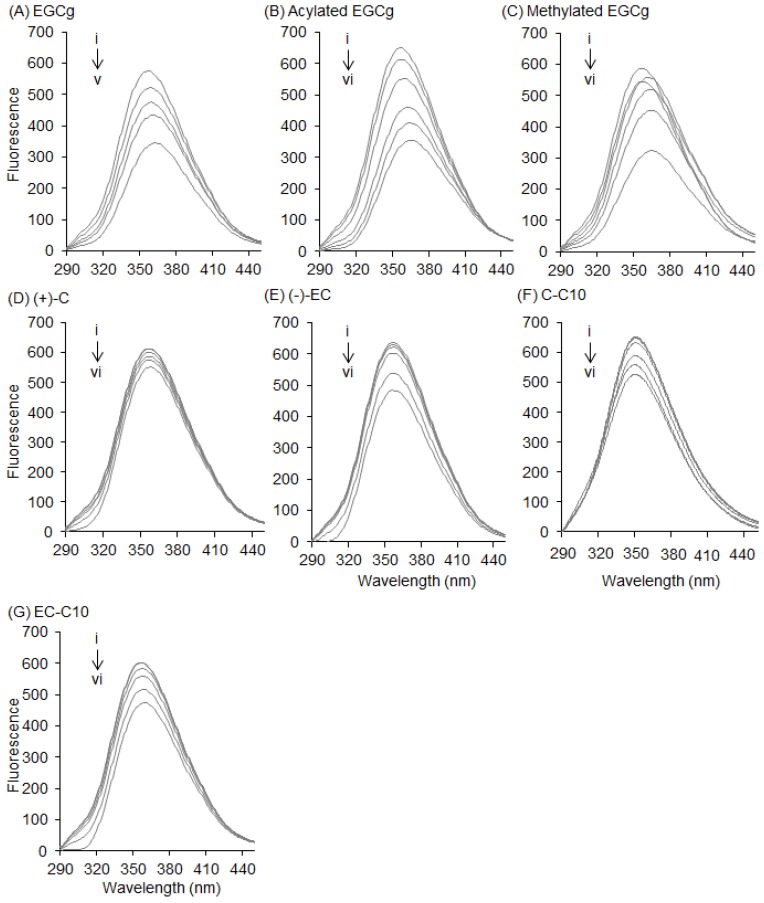
Effects of catechins and their derivatives on the fluorescence of human serum albumin (HSA). Fluorescence spectra of 0.3 mg/mL HSA with various concentrations of (**A**) EGCg; (**B**) acyl-EGCg; (**C**) methyl-EGCg; (**D**) (+)-C; (**E**) (−)-EC; (**F**) C-C10; and (**G**) EC-C10 were measured at an excitation wavelength of 280 nm. Symbols i to vi show the spectra of HSA with increasing quencher concentrations of 0, 1, 5, 12.5, 25, and 50 µM, respectively.

Finally, we investigated the interaction between HSA and catechins and their derivatives (Figure 6). HSA has one Trp residue (Trp-214) located in drug-binding site I, and this residue behaves similarly to the Trp-213 of BSA [22]. EGCg and its derivatives decreased the fluorescence intensity of HSA in a dose-dependent manner (Figure 6A–C), and the fluorescence peak wavelength was red-shifted, like that of BSA. The natural catechins and 3-*O*-acyl-catechins also decreased the fluorescence intensity of HSA in a dose-dependent manner, but these effects were smaller than in BSA (Figure 6D–G). Moreover, shift of the fluorescence peak wavelength against HSA was weaker than that against BSA. 

## 3. Discussion

Flavonoids are known to interact with the Trp residue in the drug-binding site of serum albumin [23,24]. In this study, we directly observed the interaction between EGCg and BSA in a docking simulation (Figure 1). In this model, a gallate ester moiety (D-ring) at the C-3 position enhanced the binding ability of catechins to BSA by interacting with both Trp-134 and Trp-213 by π–π stacking (Figure 1B,E). To our knowledge, this is the first reported docking simulation of EGCg and serum albumin. By Stern–Volmer analysis of the fluorescence quenching of a set of catechins, we confirmed that all the catechins interacted with albumin at the same binding site. EGCg had the strongest binding affinity against serum albumin among eight natural catechins according to their Kq values (Table 1). Moreover, the addition of an acyl group at the C-3 position of the catechins also enhanced the binding affinity to BSA and HSA (Figure 3, Figure 5, and Figure 6, and Table 1). Thus, the C-3 position of catechins plays an important role in binding to proteins, including serum albumin.

In the current study, EGCg caused a red shift of the peak wavelength in a dose-dependent manner (Figure 3A), and this tendency was also observed in other gallate-type catechins (data not shown). On the other hand, non-gallate-type catechins did not cause a shift of the peak wavelength. It was reported that the glycosides of kaempferol and kaempferide caused fluorescence quenching of BSA without a shift of the peak wavelength [25]. Together, these results imply that the gallate moiety of the catechin structure is responsible for the red shift of the peak wavelength. Moreover, our docking study demonstrated that EGCg interacted with Trp-213 in drug-binding site I by π–π stacking (Figure 1F). It is known that warfarin, ibuprofen, and digitoxin bind to drug-binding sites I, II, and III, respectively [26,27,28]. Warfarin also decreases the fluorescence of serum albumin and causes a red shift of the peak wavelength (Figure S1). Although warfarin does not have a gallate moiety, it does have a phenyl group. This phenyl group may be responsible for the red shift of the peak wavelength. Together, these results strongly suggest that gallate-type catechins bind to the drug-binding site I of serum albumin and cause a red shift of the peak wavelength in the same way as warfarin.

The Kq value of EGCg against BSA was much higher than that against HSA (Table 1), implying that it interacted more strongly with the Trp-134 residue at the molecular surface of BSA than with the Trp-213 residue at drug-binding site I (both interactions shown in Figure 1). In the folded structures of proteins, including albumins, hydrophobic amino acids are generally embedded in cylinders, forming hydrophobic cavities [29]. Since EGCg is hydrophilic, it can access the Trp residue at the surface of BSA more easily than the residue at drug-binding site I. Thus, EGCg mainly interacts with BSA via the surface Trp residue.

Substitution of the methyl group by a hydroxyl group on the D-ring of EGCg gave a lower Kq value compared with the parent compound, EGCg. On the other hand, substitution by acyl group(s) increased the Kq value. The mechanism by which these substitutions altered the Kq values remains unclear. It cannot be deduced from our results, although we speculate that these substitutions influenced the binding of the compounds with the Trp residues. Further experiments are needed to clarify this issue in future.

Our results showed that 3-*O*-acyl-catechins strongly quenched the fluorescence intensity of BSA, with a blue shift of the fluorescence peak wavelength, and that the optimal chain length of the acyl group for quenching was C10 (Figure 5). We propose the following mechanism for the blue shift. The acyl group alters the environment around the Trp residue in BSA such that the BSA–catechin interaction is heavily weakened. We speculate that the hydrophobic acyl chains surround the Trp residue in a pillar formation, isolating this residue from its environment. Then, an excited electron in the Trp residue interacts with phonons in the acyl groups, causing fluorescence quenching and the blue shift of the peak wavelength [30,31]. The existence of an optimal chain length of the acyl chain for quenching BSA indicates that longer or shorter chains interact less strongly with the Trp residue. However, it is unclear why the interaction with the Trp residue is maximized at the acyl chain length of C10. Further experiments are needed to clarify this issue. We found that acyl substitution did not affect the ability of catechins to quench the fluorescence of HSA (Figure 6). This result supports the hypothesis that catechins mainly interact with the surface of serum albumin via the C-3 position, and that this position determines their binding affinity against serum albumin. 

## 4. Material and Methods

### 4.1. Chemicals

BSA (F-V) and HSA (F-V) were purchased from Nacalai Tesque (Kyoto, Japan). Acylated EGCg (purity: ca. 98%) was synthesized by lipase-catalyzed transesterification according to a method reported previously [32]. EGCg (≥98%) and methylated EGCg (≥98%) were purchased from Nagara Science (Gifu, Japan). The acylated catechin and epicatechin derivatives (≥95%)—which are conjugated fatty acids—were chemically synthesized as described previously [33]. These structures are shown in Figure 2.

### 4.2. Docking Simulation between EGCg and BSA

To better understand the binding modes of EGCg to BSA at the atomic level, we performed molecular docking studies. Docking of the minimized-energy structure of EGCg into the crystal structure of BSA in complex with 3,5-diiodosalicylic acid (PDB code 4JK4) was carried out using the docking program of the Molecular Operating Environment (MOE) suite [34]. The prediction of the binding sites performed by the MOE Site Finder module confirmed the binding sites defined by the co-crystallized ligand in the crystal structure of BSA [35]. The docking study was performed by the default Triangle Matcher placement method. To rank the final poses, the GBVI/WSA dG scoring function—which estimates the free energy of binding of the ligand from a given pose—was used. From the three lowest S scores in the output databases of MOE docking, the ligand–BSA complex for each ligand was manually selected [36]. The docking workflow followed the “induced fit” protocol, in which the side chains of the receptor pocket were allowed to move according to the ligand conformations of EGCg, with a constraint on their positions. The weight used for tethering side chain atoms to their initial positions was 10. Prior to docking, the force field AMBER10:EHT and the implicit solvation model Reaction Field (R-field) were selected [37]. 

### 4.3. Analysis of Fluorescence Quenching of BSA and HSA

Analysis of fluorescence quenching was performed as described previously [20]. A series of mixtures was prepared with catechins or their derivatives at various concentrations (as indicated in each figure legend) and a fixed concentration of BSA (0.3 mg/mL). After the mixture was incubated at 20 °C for 20 min, the fluorescence spectrum of BSA in the mixture was measured at 20 °C in 50 mM sodium phosphate buffer (pH 7.0) at an excitation wavelength of 280 nm and with emission in the range 290–450 nm. All measurements were performed in triplicate. Fluorescence quenching data were analyzed by the Stern–Volmer equation (Equation (1)) [21]:

F_0_ = F (1 + Kq × Q), (1)

where F_0_ and F are the relative fluorescence intensities of BSA without and with catechin, respectively, Kq is the Stern–Volmer quenching constant, and Q is the concentration of catechin.

### 4.4. Statistical Analysis

The data in Table 1 and Figure 5G are expressed as means ± SE from at least triplicate independent analyses. Statistical analysis of the data in Figure 5G was performed by Dunnett’s test using JMP statistical software version 11.2.0 (SAS Institute, Cary, NC, USA), and the level of significance was set at *p* < 0.05.

## 5. Conclusions

We investigated the interaction of catechins and their derivatives with BSA or HSA using docking simulations and fluorescence quenching analysis. The results indicate that the C-3 position of the catechin structure plays an important role in the binding affinity of catechins against serum albumin. The findings also suggest that fluorescence analysis could serve as a simple and high-throughput screening method to estimate the interaction between a food factor and its target protein, although another method, such as docking, is needed to confirm the mechanism of interaction. 

## Figures and Tables

**Table 1:** Kq values of catechins against BSA and HSA.

Coumpunds	BSA	HSA
(+)-C	0.005 ± 0.0003	0.002 ± 0.0001
Cg	0.034 ± 0.0092	0.011 ± 0.0011
GC	0.001 ± 0.0001	0.001 ± 0.0001
GCg	0.019 ± 0.0030	0.009 ± 0.0008
(−)-EC	0.005 ± 0.0011	0.006 ± 0.0013
ECg	0.060 ± 0.0118	0.007 ± 0.0011
EGC	0.001 ± 0.0003	0.001 ± 0.0002
EGCg	0.225 ± 0.0192	0.033 ± 0.0065
Acyl-EGCg	0.286 ± 0.0323	0.023 ± 0.0055
Methyl-EGCg	0.150 ± 0.0109	0.014 ± 0.0021

## Supplementary Materials

The following are available online, Figure S1: Effect of drug-binding site-specific chemicals on fluorescence of BSA.

## Abbreviations

The following abbreviations are used in this manuscript:

BSA: bovine serum albumin
HSA: human serum albumin
EGCg: epigallocatechin gallate
(+)-C: catechin
(−)-EC: epicatechin
Trp: tryptophan
