# Supplementary material for: Substitution at the C-3 Position of Catechins Has an Influence on the Binding Affinities against Serum Albumin
Source: Molecules. 2017 Feb 18;22(2):314. doi: 10.3390/molecules22020314 (PMC6155608; doi:10.3390/molecules22020314)
Supplement: Supplementary file 1 [file molecules-22-00314-s001.pdf]

Supplementary Materials: The C-3 Position of Catechins Regulates their Binding Affinities against Serum Albumin

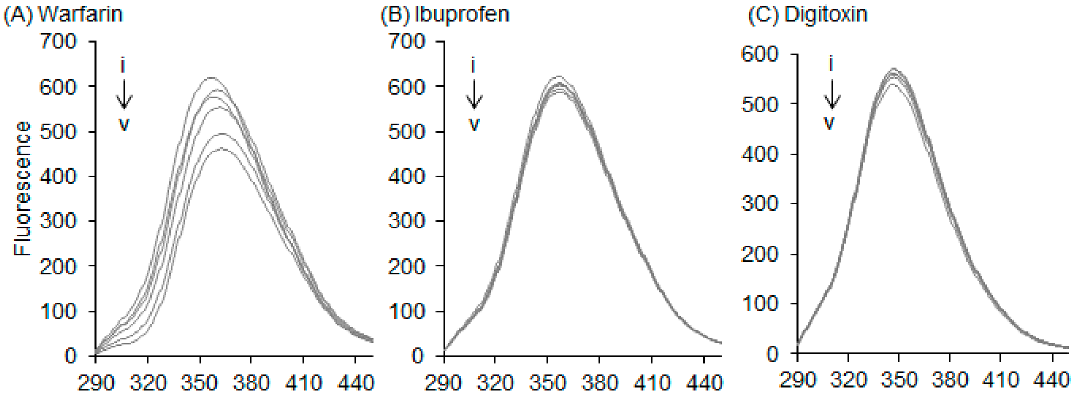

Figure S1. Effect of drug binding site specific-chemicals on fluorescence of BSA. Fluorescence spectrum of 0.3 mg/mL BSA with various concentrations of warfarin (**A**) for drug-binding site I, ibuprofen (**B**) for site II and digitoxin (**C**) for site III was measured with an excitation wavelength at 280 nm. Symbols i, ii, iii, iv, and v show the spectrum of BSA with 0, 1, 5, 12.5, 25, and 50 μM compounds, respectively.
